# Identifying Potassium-Associated Ecological Transition Zones for *Mimosa pigra* Invasion Using Explainable Machine Learning and Bayesian Inference

**DOI:** 10.3390/biology15151314

**Published:** 2026-08-06

**Authors:** Bhuvadol Gomontean, Aljo Clair Pingal, Khemmanant Khamthong

**Affiliations:** 1 Department of Biology, Faculty of Science, Mahasarakham University, Maha Sarakham 44150, Thailand; bhuvadol.g@msu.ac.th; 2 Department of Mathematics and Statistics, Mindanao State University-Iligan Institute of Technology, Iligan City 9200, Philippines; aljoclair.pingal@g.msuiit.edu.ph; 3 Department of Mathematics, Faculty of Science, Mahasarakham University, Maha Sarakham 44150, Thailand

**Keywords:** explainable machine learning, bayesian modeling, *Mimosa pigra*, invasive species, soil potassium thresholds, SHAP interpretation

## Abstract

Understanding nonlinear ecological responses is essential for improving the prediction and management of biological invasions. This study presents an integrated analytical workflow combining explainable artificial intelligence (XAI) and Bayesian inference to investigate the influence of soil potassium on *Mimosa pigra* invasion in the Chi River Basin, Thailand. Using field observations from 50 sampling plots, XGBoost characterized nonlinear relationships between environmental variables and invasion density, whereas TreeSHAP quantified predictor contributions and identified model-derived ecological transition zones from SHAP response curves. Bootstrap resampling was used to evaluate the stability of the estimated transition zone. The results revealed a pronounced nonlinear association between soil potassium availability and predicted invasion density, with a transition zone occurring near 34 ppm and the strongest positive model contributions occurring around 45 ppm. Bayesian generalized linear models quantified statistical uncertainty and compared Poisson and Negative Binomial count models, with the Poisson model providing the best overall fit. Nested five-fold cross-validation indicated consistent internal predictive performance, although independent external validation is required to assess generalizability before broader ecological application. Overall, the integrated XAI–Bayesian workflow provides an interpretable and reproducible strategy for detecting nonlinear ecological responses, quantifying uncertainty, and generating data-driven hypotheses to support invasive plant monitoring and evidence-based ecosystem management.

## Simple Summary

*Mimosa pigra* is one of the most aggressive invasive plants affecting wetland ecosystems in Thailand and many tropical regions. Understanding the environmental conditions that promote its spread is essential for improving ecological monitoring and management. In this study, we combined explainable artificial intelligence (XAI) and Bayesian statistical modeling to investigate how soil potassium influences *Mimosa pigra* invasion. Our analyses identified a nonlinear relationship between soil potassium availability and invasion intensity, revealing a model-derived ecological transition zone near 34 ppm. By integrating machine learning with statistical inference, the proposed framework provides interpretable insights into environmental drivers while explicitly quantifying uncertainty. Although validation using independent datasets is still required, this approach offers a transparent and reproducible strategy for supporting invasive species monitoring and generating ecological hypotheses for future research.

## 1. Introduction

Invasive alien plant species (IAPS) are among the leading drivers of global biodiversity loss and ecosystem degradation, particularly in vulnerable wetland ecosystems where they disrupt ecological processes and diminish ecosystem services [1,2]. One of the most aggressive wetland invaders is *Mimosa pigra* L. (giant sensitive plant), a nitrogen-fixing woody shrub native to the Neotropics that has become widely established throughout the floodplains of Southeast Asia. Its invasion success is characterized by rapid biomass accumulation, prolific seed production, and the formation of dense monospecific stands that suppress native vegetation, alter habitat structure, and reduce ecosystem resilience [3,4]. In Thailand, particularly within the Chi River Basin, the continued expansion of *M. pigra* has become an important ecological and hydrological concern because dense infestations obstruct natural water flow, complicate irrigation management, and impair wetland ecosystem functioning.

The establishment and spread of *M. pigra* are regulated by environmental drivers operating across multiple spatial scales. While climatic conditions largely determine broad-scale habitat suitability, local edaphic characteristics govern plant establishment, growth, and population density within individual sites [5]. Soil physicochemical properties, including nutrient availability, texture, and water-holding capacity, influence resource acquisition and competitive performance, thereby affecting invasion success. Previous field investigations in the Chi River Basin demonstrated that soil nutrient composition, particularly extractable potassium, was significantly associated with spatial variation in *M. pigra* density [6]. These findings suggest that potassium availability may play an important role in regulating invasion intensity, although the underlying ecological response patterns remain insufficiently understood.

Among the essential macronutrients, potassium plays a distinctive physiological role because it regulates osmotic balance, enzyme activation, carbohydrate transport, stomatal function, and plant responses to environmental stress [7,8]. For nitrogen-fixing woody legumes such as *M. pigra*, adequate potassium availability is particularly important because biological nitrogen fixation is an energy-intensive process that requires efficient photosynthate transport and metabolic regulation [8]. Consequently, variation in soil potassium availability may substantially influence plant establishment, biomass production, and competitive performance, making potassium a biologically meaningful environmental predictor for investigating invasion dynamics.

Although previous studies have identified significant associations between soil properties and *M. pigra* density, most have relied on conventional regression approaches that primarily estimate average linear effects across environmental gradients. Such methods are less capable of identifying nonlinear responses or transition zones where relatively small environmental changes correspond to disproportionately large changes in invasion intensity. Ecological systems frequently exhibit threshold-like behavior, whereby gradual environmental variation may trigger abrupt shifts in ecosystem processes or species performance [9,10]. Identifying these nonlinear transition zones is therefore important for improving ecological understanding and providing early-warning indicators that may support invasive species monitoring and management.

Recent advances in explainable artificial intelligence (XAI) have substantially improved the interpretability of machine-learning models while retaining their ability to capture complex nonlinear relationships [11,12,13]. In ecological research, XAI methods have increasingly been employed to quantify predictor contributions and reveal nonlinear environmental responses that are difficult to detect using conventional statistical approaches. Bayesian statistical models, in contrast, provide rigorous probabilistic inference, explicit uncertainty quantification, and interpretable parameter estimation. However, relatively few ecological studies have explicitly integrated explainable machine learning with Bayesian inference within a unified analytical framework for investigating invasive plant dynamics. Combining these complementary methodologies offers the potential to improve predictive performance while simultaneously enhancing ecological interpretation and statistical inference.

To bridge the gap between predictive capability and statistical inference, this study proposes an integrated analytical framework that combines XGBoost, TreeSHAP, bootstrap uncertainty analysis, and Bayesian count regression. Tree-based ensemble algorithms such as XGBoost effectively capture complex nonlinear interactions among environmental variables [14], whereas TreeSHAP decomposes model predictions into additive feature contributions, thereby enabling the transparent interpretation of predictor effects and nonlinear response patterns [11,12,13]. Because SHAP values quantify local feature contributions rather than direct ecological thresholds, model-derived transition zones were identified from LOESS-smoothed SHAP response curves. Bootstrap resampling was subsequently applied to assess the stability and uncertainty associated with the estimated transition zones [15].

To complement machine-learning feature discovery with formal statistical inference, Bayesian count regression models were employed to evaluate the associations between environmental variables and *M. pigra* density. Because ecological count data may exhibit overdispersion, both Poisson and Negative Binomial formulations were evaluated. Bayesian estimation enables full posterior inference, formal uncertainty quantification, and objective model comparison using predictive performance criteria, thereby supporting the selection of an appropriate statistical model for the observed data [16].

In this study, we develop an integrated and reproducible analytical workflow combining XAI-driven machine learning with Bayesian count modeling to investigate the edaphic controls of *M. pigra* invasion in the Chi River Basin. Specifically, the objectives are to (1) identify nonlinear, model-derived transition zones of soil potassium associated with changes in predicted *M. pigra* density; (2) evaluate how soil physical properties influence potassium-associated invasion patterns; and (3) assess the utility of the integrated XAI–Bayesian workflow for ecological prediction, uncertainty quantification, and hypothesis generation.

By integrating the predictive capabilities of ensemble learning with the inferential strengths of Bayesian statistics, this study provides a transparent and reproducible analytical workflow for identifying model-derived ecological transition zones while explicitly quantifying uncertainty. Rather than establishing definitive ecological thresholds, the proposed workflow is intended to generate interpretable, data-driven hypotheses that can guide future ecological validation and support evidence-based management of invasive plant species.

The remainder of this article is organized as follows: Section 2 describes the study area, field sampling strategy, and analytical workflow. Section 3 presents the model results and performance evaluation. Section 4 discusses the ecological implications and methodological contributions, and Section 5 summarizes the principal findings.

## 2. Materials and Methods

All statistical analyses were performed using R version 4.4.1 (R Foundation for Statistical Computing, Vienna, Austria). Machine-learning analyses were conducted using the R package xgboost (version 1.7.8.1), and TreeSHAP explanations were generated using the R package SHAPforxgboost (version 0.2.0). Bayesian analyses were performed using the R package brms (version 2.22.0) with the cmdstanr interface (version 0.8.0) and CmdStan (version 2.38.0).

### 2.1. Data Source and Sampling Design

This study utilizes a field dataset originally reported by [6], which investigated the environmental drivers of *Mimosa pigra* L. distribution within riparian ecosystems of the Chi River Basin, Maha Sarakham Province, Thailand. Sampling sites were selected using a stratified design to capture variability across representative habitat conditions, including differences in flood exposure, vegetation structure, and disturbed areas surrounding wetlands affected by topsoil displacement (land filling), thereby ensuring coverage across key ecological gradients.

Field surveys were conducted across three districts, Mueang, Kantharawichai, and Kosum Phisai, comprising 25 sampling locations. At each site, two 2×2 m plots were established, resulting in a total of 50 sampling plots. This design enabled the robust characterization of spatial heterogeneity while maintaining consistency in sampling effort.

**Figure 1:**
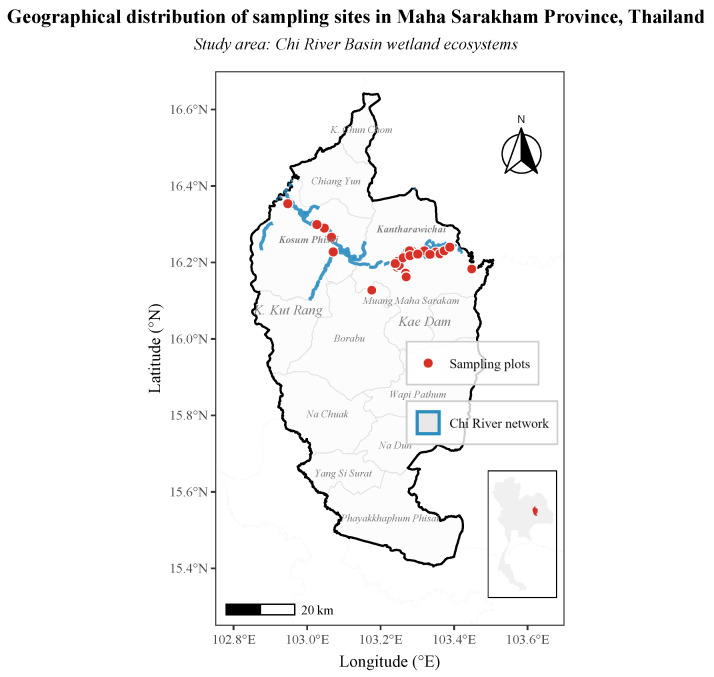
Study area and spatial distribution of sampling locations in the Chi River Basin, Maha Sarakham Province, Northeastern Thailand. The main map illustrates the locations of 25 sampling sites (red dots) distributed along the Chi River network (blue lines). At each site, two 2×2 m sampling plots were established, yielding a total of 50 sampling plots that represent a range of riparian and wetland environments. These sites were selected using a stratified sampling design to capture variability in flood exposure, vegetation structure, and disturbance conditions across the study area. The inset map shows the geographical location of the study area within Thailand.

Within each plot, the total number of *M. pigra* individuals was recorded and used as a count-based response variable, expressed as individuals per 4 m^2^. The spatial configuration of sampling plots is illustrated in Figure 1.

Although the dataset comprised 50 sampling plots, the primary objective of this study was to identify nonlinear environmental response patterns and generate interpretable ecological hypotheses rather than to develop a large-scale predictive model. To reduce the risk of overfitting associated with a relatively small dataset, subsequent analyses incorporated repeated cross-validation, bootstrap resampling, and Bayesian uncertainty quantification. These complementary approaches were intended to assess the internal consistency and statistical stability of the proposed analytical framework within the observed environmental gradients.

### 2.2. Field Sampling and Soil Analysis

Soil samples were collected from the upper 0–30 cm layer, representing the biologically active horizon that strongly influences nutrient availability and seedling establishment. At each sampling plot, composite soil samples were obtained by combining multiple subsamples to minimize local heterogeneity and improve representativeness. This composite sampling strategy was adopted to reduce microscale spatial variability and to obtain representative estimates of soil physicochemical properties for each sampling plot.

Soil texture fractions (sand, silt, and clay) were quantified using the hydrometer method (with reagents sourced from Merck) and classified according to the USDA soil classification system, following field and laboratory protocols described in the Soil Survey Manual [17]. This approach ensured consistency with internationally recognized soil classification standards.

Available phosphorus was determined using the Bray II extraction method [18], utilizing analytical-grade chemical substances obtained from Merck. Exchangeable potassium (K) concentrations (ppm) were measured using the ammonium acetate extraction method, followed by analysis via flame photometry and atomic absorption spectrometry using an Atomic Absorption Spectrometer (Analytik Jena, ZEEnit 700P series) [19]. These analytical procedures were conducted in accordance with standard soil analysis protocols to ensure consistency and comparability with previous studies.

All laboratory analyses were performed under established quality control procedures, including calibration with standard reference materials and duplicate measurements, to ensure analytical accuracy and reliability.

### 2.3. Methodological Rationale

Conventional regression models provide interpretable estimates of average effects but are generally limited in their ability to capture complex nonlinear interactions among environmental variables. Conversely, machine-learning algorithms such as XGBoost effectively model nonlinear relationships and higher-order interactions but often lack ecological interpretability because of their black-box nature. Explainable artificial intelligence (XAI), particularly TreeSHAP, addresses this limitation by decomposing model predictions into additive feature contributions, thereby enabling the interpretation of predictor effects and identification of model-derived transition zones. Bayesian regression models complement machine-learning approaches by providing probabilistic parameter estimation, explicit uncertainty quantification, and formal model comparison. Integrating XAI with Bayesian inference therefore combines the strengths of predictive modeling and statistical inference, providing a transparent framework for investigating nonlinear ecological responses while appropriately characterizing uncertainty.

### 2.4. Overall Analytical Framework

**Figure 2:**
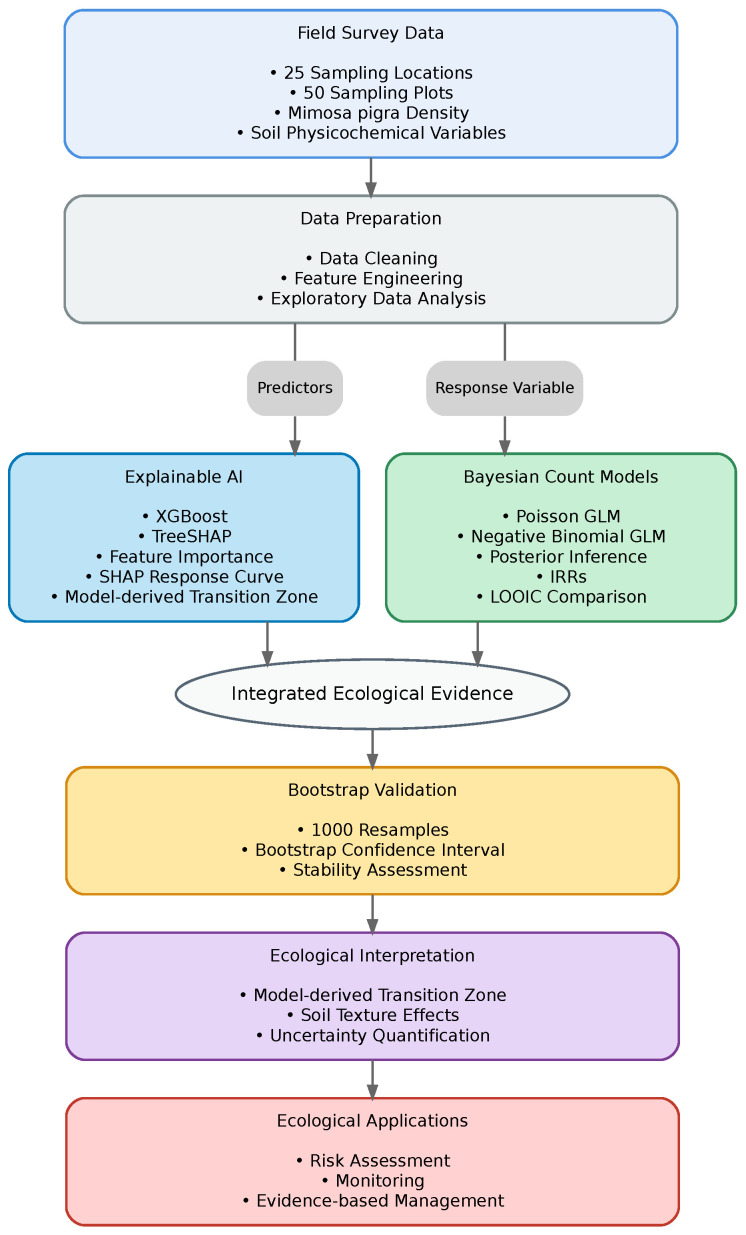
Overall analytical workflow of the proposed hybrid XAI–Bayesian framework for identifying potassium-associated ecological transition zones in *Mimosa pigra* invasion. Field survey data were analyzed using two complementary approaches: explainable artificial intelligence (XAI) based on XGBoost and TreeSHAP to characterize nonlinear predictor contributions and model-derived transition zones, and Bayesian count models to quantify uncertainty, estimate incidence rate ratios (IRRs), and compare alternative model formulations using LOOIC. Bootstrap resampling was applied to evaluate the stability of the identified transition zone. The integrated outputs provide an interpretable framework for ecological inference, uncertainty assessment, and invasive species management applications.

The overall analytical workflow of the proposed hybrid XAI–Bayesian framework is illustrated in Figure 2. The framework consists of seven sequential components. First, field survey data, including *M. pigra* density and soil physicochemical variables, were collected from 25 sampling locations (50 sampling plots) in the Chi River Basin. Second, the dataset was preprocessed through data cleaning, feature engineering, and exploratory data analysis to ensure data quality and consistency. Third, XGBoost was employed to characterize nonlinear relationships between environmental variables and *M. pigra* density, while TreeSHAP was used to quantify feature contributions and identify model-derived transition zones associated with soil potassium. Fourth, Bayesian Poisson and Negative Binomial count models were fitted to quantify statistical uncertainty, estimate incidence rate ratios (IRRs), and compare alternative model specifications using the leave-one-out information criterion (LOOIC). Fifth, bootstrap resampling (1000 replications) was performed to evaluate the statistical stability of the estimated transition zone and its associated confidence interval. Finally, the complementary results from the explainable machine learning and Bayesian analyses were integrated to provide an interpretable assessment of nonlinear ecological responses, uncertainty quantification, and their potential applications for ecological risk assessment and wetland management.

### 2.5. Statistical Modeling and Machine Learning Framework

Building upon the Bayesian count modeling framework of [6], this study implements a hybrid analytical approach to capture both nonlinear ecological responses and statistical uncertainty. XGBoost was employed to model complex interactions and nonlinear relationships, with hyperparameter tuning conducted using cross-validation to optimize predictive performance.

Model interpretability was achieved using SHAP, implemented via the TreeSHAP algorithm, to quantify variable contributions and identify potential ecological thresholds.

To complement the machine learning framework, Bayesian generalized linear models (GLMs) with both Poisson and Negative Binomial likelihoods were initially considered to account for potential overdispersion in count data and to quantify parameter uncertainty. Weakly informative priors were specified, and model convergence was assessed using standard diagnostics (e.g., $\hat{R}$ and effective sample size).

All analyses were conducted in R (version 4.4.1), and the full code and workflow are available in a public repository to ensure reproducibility and transparency.

#### 2.5.1. Predictor Variables

The predictor variables used in the modeling framework were selected based on their ecological relevance to plant growth and invasive species establishment in floodplain environments. Soil potassium and phosphorus (ppm) represent key macronutrients influencing plant physiological processes, while soil pH and bulk density reflect important soil chemical and physical properties that regulate nutrient availability and root development. Structural plant attributes, including stem diameter and plant height, were included as indicators of stand development and competitive dominance. These variables were therefore chosen to capture both the edaphic conditions and population structure that may influence the density dynamics of *M. pigra*.

#### 2.5.2. Bayesian Inferential Framework

To evaluate the influence of soil potassium while accounting for environmental and biological covariates, both Bayesian Poisson and Negative Binomial (NB) regression models were initially considered. The negative binomial formulation was included to accommodate potential overdispersion in count data. Model comparison was performed using Leave-One-Out cross-validation (LOO) and the corresponding Leave-One-Out Information Criterion (LOOIC), which assesses out-of-sample predictive performance. Based on this comparison, the Poisson model demonstrated superior predictive performance and was therefore selected as the final inferential model for subsequent analysis.

The selected model was implemented using the brms package with the cmdstanr interface and the CmdStan backend, which performs Hamiltonian Monte Carlo (HMC) sampling via the Stan probabilistic programming framework.

Let $Y_{i}$ denote the observed number of *M. pigra* individuals in plot *i*. The response variable was modeled as

(1) $$Y_{i}∼\mathrm{Poisson}\left(\mu_{i}\right)$$

The expected value $\mu_{i}$ was linked to the predictors through a log-link function:

(2) $$\log\left(\mu_{i}\right)=\beta_{0}+\beta_{1}K_{i}+\beta_{2}T_{i}+\beta_{3}D_{i}$$

where $K_{i}$ represents exchangeable soil potassium concentration, $T_{i}$ denotes soil texture (treated as a categorical factor), and $D_{i}$ corresponds to plant diameter. These predictors were selected based on their ecological relevance and their observed contributions in the preliminary machine-learning analyses.

Weakly informative priors were specified to regularize parameter estimation:

- Regression coefficients ($\beta_{j}$): $\beta_{j}∼\mathrm{Normal}\left(0,1\right)$ for j=1,…,3.
- Intercept ($\beta_{0}$): $\beta_{0}∼\mathrm{Student}-t\left(3,0,5\right)$.

Posterior distributions were estimated using Hamiltonian Monte Carlo (HMC) sampling. Four Markov chains were run with 4000 iterations each, including 2000 warm-up iterations, resulting in 8000 post-warm-up samples. No divergent transitions were detected during sampling. Convergence was assessed using the potential scale reduction factor ($\hat{R}$), with all parameters exhibiting values below 1.01. Adequate sampling efficiency was further confirmed through effective sample size diagnostics and visual inspection of trace plots.

#### 2.5.3. Extraction of Ecological Thresholds and Optima

To quantify the nonlinear influence of soil potassium on invasion dynamics, two ecological indicators were derived from the SHAP response curves generated by the XGBoost model.

##### Critical Invasion Threshold ($K_{\mathrm{threshold}}$)

The critical threshold was defined as the potassium concentration at which the SHAP contribution transitions from negative to positive, indicating a shift from a limiting to a positive influence on predicted invasion density. Formally, this point corresponds to the first intersection of the SHAP response function with the zero baseline:

(3) $$K_{\mathrm{threshold}}=\min\left\{K:\mathrm{SHAP}\left(K\right)=0\right\}$$

This threshold represents the potassium concentration at which soil nutrient availability begins to exert a positive contribution to the predicted density of *Mimosa pigra*.

##### Ecological Optimum ($K_{\mathrm{optimum}}$)

The ecological optimum was defined as the potassium concentration associated with the maximum SHAP contribution along the observed potassium gradient:

(4) $$K_{\mathrm{optimum}}=\arg\max_{K}\mathrm{SHAP}\left(K\right)$$

This value represents the nutrient level at which potassium exerts the strongest positive contribution to the predicted population density of *M. pigra*.

To assess whether the machine learning-derived threshold patterns were consistent with statistical inference, the identified threshold regions were qualitatively compared with posterior estimates obtained from the selected Bayesian Poisson model. Because the Bayesian model did not explicitly estimate threshold values, it was used to evaluate the overall direction and magnitude of the potassium effect rather than to confirm a specific breakpoint.

This complementary analysis allowed assessment of whether the nonlinear response patterns identified by the XGBoost–SHAP framework were broadly consistent with the posterior trends estimated under the Bayesian inferential model.

#### 2.5.4. Bootstrap Uncertainty Assessment

To evaluate the robustness of the identified potassium threshold, a nonparametric bootstrap procedure was performed. The original dataset was resampled with replacement 1000 times. For each bootstrap sample, the XGBoost model was refitted, SHAP values were recalculated, and the threshold value was re-estimated using the SHAP zero-crossing criterion.

The empirical distribution of bootstrap threshold estimates was summarized using the mean, median, and 95% percentile confidence interval. This procedure provided an uncertainty assessment for the estimated ecological threshold and allowed evaluation of its stability across resampled datasets.

#### 2.5.5. Sensitivity Analysis

Because threshold identification depends partly on the smoothing procedure applied to the SHAP response curve, a sensitivity analysis was conducted using multiple LOESS smoothing spans (0.50, 0.75, and 1.00). Threshold estimates obtained under alternative smoothing specifications were compared to evaluate the stability of the inferred potassium transition point.

Consistent threshold estimates across smoothing settings were interpreted as evidence of robustness in the identified nonlinear response pattern.

#### 2.5.6. Cross-Validation Procedure

To evaluate model robustness and prevent overfitting, a *k*-fold cross-validation procedure was implemented. The dataset was randomly partitioned into k=5 folds, where each fold was used once as a validation set while the remaining folds were used for training.

For each fold, predictive accuracy was evaluated by comparing observed and predicted plant densities. The final model performance was reported as the average across all folds. To avoid optimistic performance estimates, predictions for each observation were generated only from models that were trained without that observation (out-of-fold predictions).

Predictive performance was summarized using the mean RMSE, MAE, and coefficient of determination ($R^{2}$) calculated from the aggregated out-of-fold predictions across the five validation folds.

#### 2.5.7. Model Evaluation Metrics

Predictive performance of the machine learning models was assessed using standard regression metrics including, the Root Mean Square Error (RMSE) and the Mean Absolute Error (MAE):

(5) $$\mathrm{RMSE}=\sqrt{\frac{1}{n}\sum_{i=1}^{n}{\left(y_{i}-{\hat{y}}_{i}\right)}^{2}}$$

(6) $$\mathrm{MAE}=\frac{1}{n}\sum_{i=1}^{n}\left|y_{i}-{\hat{y}}_{i}\right|$$

where $y_{i}$ represents the observed plant density and ${\hat{y}}_{i}$ denotes the model prediction. Lower values of RMSE and MAE indicate better predictive performance.

#### 2.5.8. Bayesian Model Diagnostics

The reliability of the Bayesian models was assessed using posterior convergence diagnostics and predictive validation metrics. Because both Poisson and Negative Binomial formulations were initially considered, model comparison was performed using Leave-One-Out cross-validation (LOO).

Model convergence was evaluated using the potential scale reduction factor ($\hat{R}$), where values close to 1 indicate satisfactory chain convergence. Predictive performance was assessed using the Leave-One-Out Information Criterion (LOOIC):

(7) $$\mathrm{LOOIC}=-2\times {\mathrm{elpd}}_{loo}$$

The model with the lower LOOIC was selected for subsequent statistical inference. Pareto-smoothed importance sampling diagnostics were also examined, with Pareto *k* values below 0.7 indicating stable importance sampling estimates.

## 3. Results and Model Synthesis

### 3.1. Descriptive Statistics

Edaphic variables and *Mimosa pigra* structural characteristics recorded across the 50 survey plots are presented in Table 1. Although the sample size is moderate, it reflects the practical constraints of conducting intensive plot-based surveys within the seasonally inundated wetlands of the Chi River Basin. In such environments, dense vegetation and fluctuating flood regimes often preclude extensive sampling; nonetheless, the resulting dataset provides a representative characterization of local invasion conditions.

The sampled plots encompassed a broad environmental gradient within the Chi River floodplain, with soil potassium concentrations ranging from 21.52 to 70.33 ppm and *M. pigra* density varying from 1.50 to 35.50 plants m^−2^. This variability reflects considerable heterogeneity in soil conditions and invasion intensity across the study area, providing sufficient variability for exploring potential nonlinear relationships between soil properties and *M. pigra* density using the proposed XAI–Bayesian framework. Nevertheless, the findings should be interpreted within the scope of the present dataset, and external validation using independent datasets from other geographical regions is needed to assess their broader generalizability.

### 3.2. Bayesian Inferential Analysis and Model Performance

Model comparison indicated modest support for the Bayesian Poisson GLM relative to the negative binomial formulation (Table 2). The Poisson model achieved a slightly lower leave-one-out information criterion (LOOIC = 293.6) than the Negative Binomial model (LOOIC = 299.8; elpd difference = −3.1, se = 4.2), together with a marginally higher Bayesian $R^{2}$ (0.522 vs. 0.469) and slightly lower prediction errors (RMSE = 3.77 vs. 3.92; MAE = 2.78 vs. 2.90). Although these differences were relatively small and the standard error of the difference (se = 4.2) exceeded the point difference (−3.1), indicating no statistically definitive superiority, they consistently favored the Poisson model. Combined with the absence of substantial overdispersion, these results suggest that the simpler Poisson formulation adequately described the observed count data.

Posterior diagnostics confirmed reliable model estimation. All parameters exhibited $\hat{R}=1.00$ and large effective sample sizes (ESSs), indicating satisfactory convergence and well-mixed Markov chains (Table 3).

Posterior estimates and incidence rate ratios (IRRs) identified several environmental factors associated with variation in *Mimosa pigra* density. Soil potassium exhibited a positive association with invasion intensity (posterior mean = 0.01, 95% CrI: 0.00–0.02), corresponding to an IRR of 1.010 (95% CrI: 1.001–1.018). This estimate corresponds to an approximately 1.0% increase in the expected *M. pigra* density for every one-unit increase in soil potassium concentration, after accounting for soil texture and plant diameter.

Several soil texture classes were also associated with higher invasion densities relative to the reference category. In particular, Clay Loam (IRR = 1.786; 95% CrI: 1.302–2.391) and Silty Clay (IRR = 1.723; 95% CrI: 1.270–2.250) exhibited the strongest positive associations. These findings are consistent with the ecological expectation that finer-textured soils, which generally possess greater water-retention capacity and nutrient availability, may provide favorable conditions for the establishment and persistence of *M. pigra* populations. These texture-specific estimates should be interpreted cautiously because several soil texture classes were represented by relatively few sampling plots, resulting in wider credible intervals for some categories.

In contrast, plant diameter was negatively associated with invasion density (IRR = 0.737; 95% CrI: 0.640–0.834). This estimate implies that a one-centimeter increase in mean plant diameter was associated with an approximately 26.3% decrease in expected density. Such a pattern may reflect density-dependent ecological processes, whereby plots dominated by larger individuals tend to contain fewer plants per unit area.

**Figure 3:**
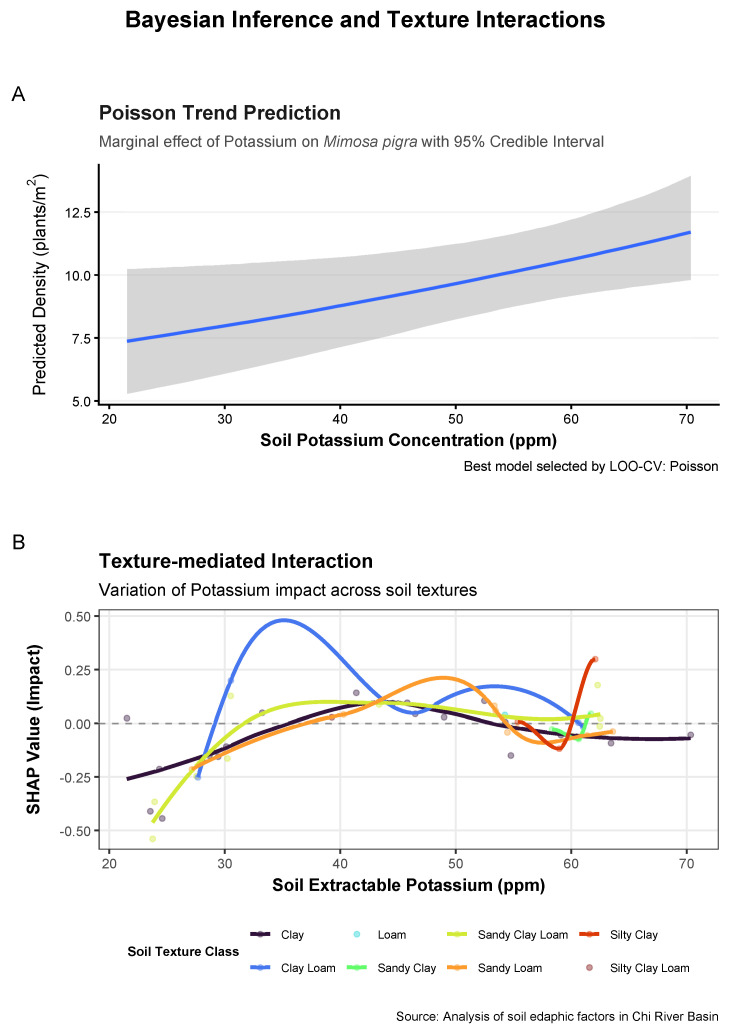
(**A**) Bayesian marginal effect of potassium on *M. pigra* density estimated from the selected Poisson regression model. The blue curve represents the posterior mean prediction, while the shaded grey band indicates the 95% credible interval (CrI). Results confirm a positive association between potassium availability and predicted invasion density. (**B**) Texture-mediated interaction illustrating how the effect of potassium varies across soil texture classes. LOESS-smoothed curves (with span = 0.75) reveal distinct response patterns among textures, indicating that soil physical properties modulate the ecological influence of potassium on invasion success.

Overall, the Bayesian analysis provides inferential evidence that potassium availability is positively associated with *M. pigra* invasion intensity, while soil texture and plant size further influence population structure. Marginal effect analysis also indicates a positive relationship between potassium availability and predicted invasion density (Figure 3A). Moreover, the texture-specific marginal effects suggest that the influence of potassium varies across soil texture classes, highlighting the potential role of soil physical properties in modulating invasion dynamics (Figure 3B). While the Bayesian model quantifies the direction and uncertainty of these associations, it assumes a parametric functional form. Therefore, the subsequent explainable machine-learning analysis using XGBoost and TreeSHAP is employed to investigate whether these relationships exhibit nonlinear response patterns, model-derived transition zones, and threshold-like behavior that may not be adequately captured by the generalized linear model.

### 3.3. Synthesis of Thresholds and Model Consensus

A key contribution of this study lies in the integration of Bayesian and machine-learning frameworks to characterize both average environmental effects and nonlinear ecological responses. The Bayesian Poisson model identified a positive association between soil potassium and *M. pigra* density after accounting for soil texture and plant diameter. In contrast, the XGBoost–SHAP framework provided finer ecological resolution by identifying nonlinear response patterns, transition zones, and regions of maximum predicted influence.

**Figure 4:**
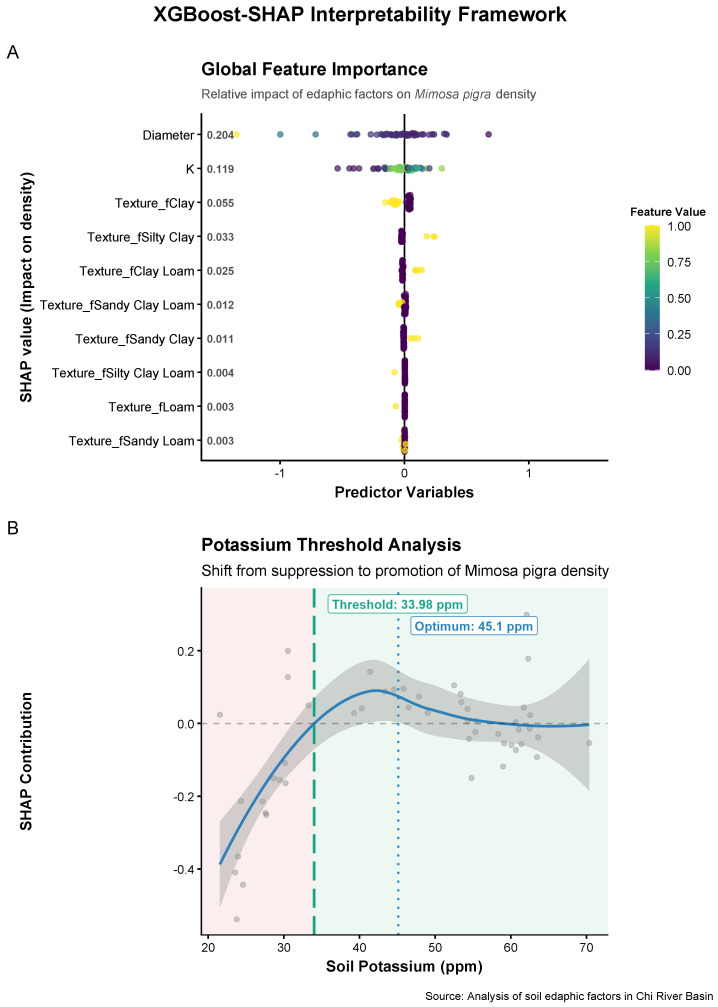
XGBoost–SHAP interpretability analysis for predicting *Mimosa pigra* density. (**A**) Colors of individual dots represent feature values. Global feature importance based on mean absolute SHAP values, ranking predictors according to their relative contribution to model output. Plant diameter exhibits the strongest influence (0.209), followed by soil potassium (0.112) and soil texture (0.066). (**B**) SHAP dependence plot illustrating the nonlinear effect of soil potassium (K) on predicted plant density. The vertical dashed line indicates the SHAP zero-crossing threshold at approximately 33.98 ppm, corresponding to the transition from a negative to a positive modeled contribution of potassium to predicted *M. pigra* density.

Feature importance analysis (Table 4) indicated that plant diameter was the most influential predictor (Gain = 0.552), followed by soil potassium (Gain = 0.281), highlighting the combined importance of structural and edaphic drivers in shaping invasion intensity (Figure 4A). Although plant diameter exhibited the highest overall predictive importance, potassium was the primary environmental variable exhibiting a pronounced nonlinear response pattern. Consequently, subsequent SHAP analyses focused on characterizing potassium-associated transition points and ecological optima (Figure 4B).

**Figure 5:**
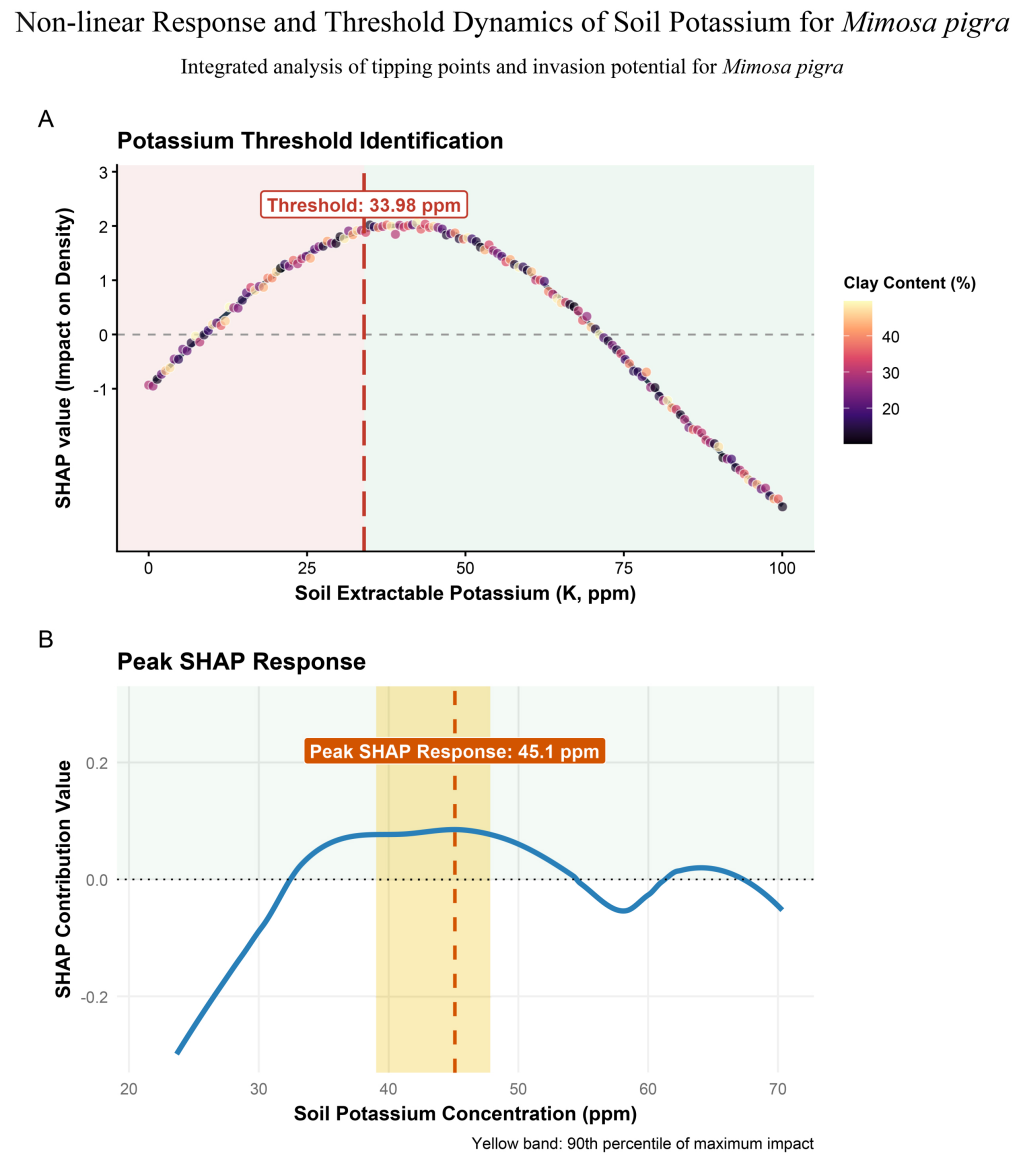
Non-linear response and threshold dynamics of soil potassium for *Mimosa pigra* invasion. (**A**) Potassium threshold identification based on SHAP analysis. The vertical dashed line indicates the SHAP zero-crossing threshold (33.98 ppm), representing the transition from a negative to a positive modeled contribution of potassium to predicted invasion density. Scatter points are colored according to clay content (%). (**B**) Peak SHAP response across the observed potassium gradient. The maximum SHAP contribution occurred at approximately 45.1 ppm, corresponding to the strongest modeled positive effect of potassium on predicted *M. pigra* density. The shaded yellow band represents the range exceeding the 90th percentile of the maximum SHAP contribution.

The SHAP response curve identified a potassium-associated transition point at approximately 33.98 ppm (Table 5 and Figure 5A), corresponding to the point at which the LOESS-smoothed SHAP response crossed zero and shifted from a negative to a positive contribution to predicted *M. pigra* density. Potassium concentrations between approximately 39.0 and 47.8 ppm were associated with the strongest positive SHAP contributions, with the maximum effect observed near 45.10 ppm (Figure 5B).

To assess the robustness of this threshold estimate, a bootstrap resampling procedure (1000 iterations) was performed. The mean bootstrap threshold was 32.40 ppm, with a 95% confidence interval of 23.88–47.64 ppm. Although this interval indicates moderate uncertainty in the precise location of the threshold, the bootstrap results support the existence of a consistent potassium-associated transition zone across resampled datasets.

Taken together, the Bayesian and explainable machine-learning results provide complementary evidence that potassium availability plays an important role in invasion dynamics. While the Bayesian model supports a positive overall association between potassium and invasion density, the SHAP analysis further suggests that this relationship is nonlinear and characterized by a transition zone near 34 ppm. These threshold estimates should be interpreted as model-derived and data-driven indicators rather than fixed ecological boundaries. Nevertheless, they provide valuable insight into potential nonlinear nutrient constraints associated with *M. pigra* invasion.

### 3.4. Soil Texture-Specific Potassium Thresholds

The relationship between potassium and *M. pigra* density was further modulated by soil texture (Table 6), indicating an interaction between nutrient availability and physical soil properties. Clay soils exhibited the lowest empirical threshold (21.52 ppm), suggesting that relatively low potassium levels may contribute positively to predicted density in fine-textured environments.

In contrast, coarser soil textures required higher potassium concentrations to achieve similar positive contributions, with thresholds increasing to 40.28 ppm in Sandy Loam and 61.68 ppm in Sandy Clay soils. This pattern may be consistent with the role of soil texture in regulating cation exchange capacity and nutrient retention.

However, it is important to note that some texture-specific estimates are derived from limited sample sizes and should therefore be interpreted with caution. Overall, these findings provide a mechanistically plausible, model-derived explanation for how soil texture may mediate the effective availability of potassium, thereby influencing invasion dynamics across heterogeneous riparian environments.

### 3.5. Model Diagnostics

**Figure 6:**
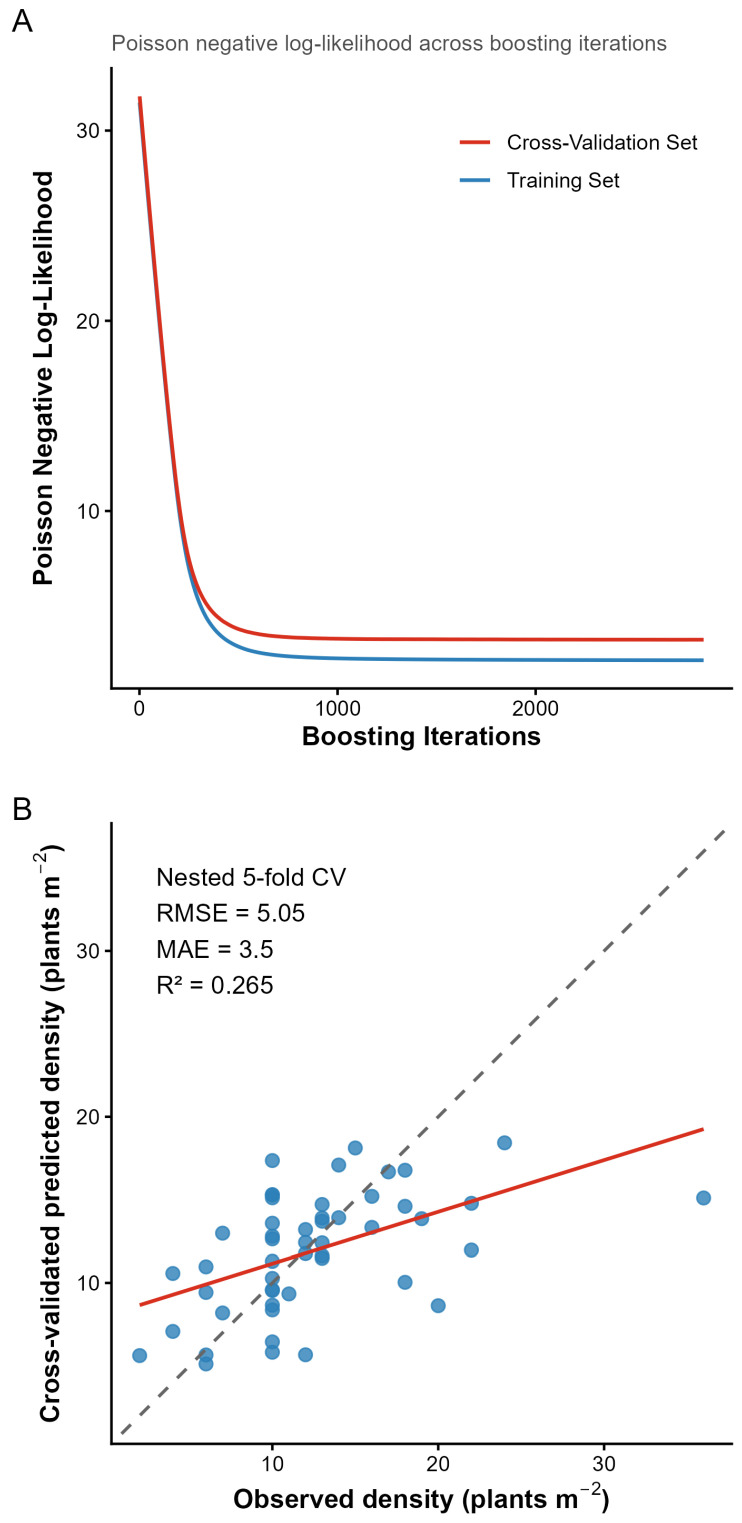
XGBoost model diagnostics. (**A**) Training and cross-validation Poisson negative log-likelihood across boosting iterations. Both curves rapidly decreased during the early stages of training and subsequently stabilized, indicating stable model convergence. (**B**) Relationship between observed and cross-validated predicted *Mimosa pigra* density obtained from nested 5-fold cross-validation. The dashed line denotes the identity line (y=x), whereas the solid line represents the fitted linear regression. Performance metrics (RMSE, MAE, and $R^{2}$) were calculated exclusively from out-of-fold predictions, providing an approximately unbiased assessment of internal predictive performance.

The predictive performance of the XGBoost model was evaluated using learning curve diagnostics and nested 5-fold cross-validation. Learning curves based on the Poisson objective function (Figure 6A) showed a rapid decline in training and test deviance during the early boosting iterations, followed by gradual stabilization. The convergence pattern between the training and cross-validation loss curves suggests that the model converged without evidence of substantial overfitting.

Predictive performance was assessed by comparing observed and cross-validated predicted *M. pigra* density using nested 5-fold cross-validation (Figure 6B), thereby reducing the risk of data leakage and overly optimistic performance estimates. The cross-validated predictions yielded a coefficient of determination ($R^{2}$) of 0.265, a root mean square error (RMSE) of 5.05 plants m^−2^, and a mean absolute error (MAE) of 3.50 plants m^−2^. These performance metrics should be interpreted in the context of ecological field datasets, where substantial biological heterogeneity, measurement uncertainty, and the relatively limited sample size (n=50) commonly constrain predictive accuracy. Nevertheless, the nested cross-validation procedure provides an approximately unbiased estimate of internal predictive performance and internal out-of-sample generalization within the available dataset.

Although the cross-validated predictive performance was moderate, these results should be interpreted in light of the inherent ecological complexity, measurement uncertainty, and relatively limited sample size (n=50). The nested cross-validation procedure ensures that the reported performance metrics reflect honest internal predictive performance rather than optimistic estimates derived from the training data.

Overall, these diagnostic results indicate that the proposed XGBoost framework provides a transparent and reproducible analytical approach for characterizing nonlinear ecological responses and identifying model-derived ecological transition zones within the observed dataset. While additional external validation using independent datasets is required to establish broader generalizability, the present results demonstrate the internal consistency and statistical robustness of the proposed framework for exploratory ecological modeling.

## 4. Discussion

### 4.1. Main Findings and Methodological Contributions

This study demonstrates that the invasion intensity of *Mimosa pigra* in the Chi River Basin is associated with local edaphic conditions, particularly soil potassium availability. By integrating explainable machine learning with Bayesian count modeling, the proposed framework provides both predictive capability and ecological interpretability. Importantly, this study extends previous work [6] by identifying nonlinear nutrient-response patterns that are not readily captured by conventional regression approaches based on linear assumptions [9,10]. Notably, the objective of the XGBoost analysis was not solely to maximize predictive accuracy but to identify interpretable nonlinear ecological response patterns and transition zones.

Unlike conventional linear regression models or black-box machine learning approaches in isolation, the integrated XAI–Bayesian framework provides a complementary approach that bridges the gap between predictive modeling and statistical inference. While traditional models fail to capture nonlinear edaphic thresholds and pure machine learning lacks probabilistic quantification of uncertainty, our dual approach simultaneously pinpoints non-parametric transition zones via SHAP and accounts for parameter uncertainty through rigorous posterior distributions.

The XGBoost–SHAP analysis revealed a model-derived potassium threshold at approximately 33.98 ppm, representing the point at which the contribution of potassium to predicted *M. pigra* density shifts from negative to positive. Potassium concentrations between approximately 39 and 48 ppm were associated with the strongest positive contributions, with the maximum SHAP effect occurring near 45.1 ppm. These findings suggest that potassium availability may influence invasion intensity in a nonlinear manner rather than through a simple linear gradient.

However, the identified threshold should be interpreted as a model-derived transition point in predicted response rather than a definitive ecological breakpoint. Bootstrap resampling (1,000 iterations) yielded a mean threshold estimate of 32.40 ppm (95% bootstrap CI: 23.88–47.64 ppm), indicating moderate uncertainty in the precise location of the transition point while supporting the overall robustness of the nonlinear response pattern. Furthermore, bootstrap-based uncertainty assessment indicated that the estimated threshold remained relatively stable across resampled datasets, suggesting that the identified transition zone is not solely an artifact of a particular sample realization. Nevertheless, uncertainty intervals should be considered when interpreting the ecological significance of the threshold estimate. The threshold reflects patterns learned from the observed data and highlights a region where invasion intensity becomes increasingly associated with potassium availability.

The Bayesian Poisson model complements these findings by supporting the overall positive relationship between potassium availability and invasion density. Although the estimated effect size was moderate, posterior inference consistently supported the importance of potassium as an environmental driver. Together, the Bayesian and XAI approaches provide complementary perspectives, combining formal statistical inference with the ability to identify complex nonlinear response patterns.

From a methodological perspective, the hybrid XAI–Bayesian framework represents a promising approach for ecological research. Machine learning algorithms such as XGBoost are highly effective at detecting nonlinear interactions but are often criticized for limited interpretability. The incorporation of SHAP values addresses this limitation by decomposing model predictions into additive feature contributions [11]. When combined with Bayesian inference, the resulting framework provides both predictive insight and statistical transparency, which are essential for ecological applications and environmental decision-making.

### 4.2. Potassium-Driven Mechanisms of Invasion Success

Potassium plays a critical role in plant physiological processes, including osmotic regulation, enzyme activation, photosynthesis, and stress tolerance. For a rapidly expanding woody invader such as *M. pigra*, sufficient potassium availability may enhance growth efficiency and biomass accumulation, thereby increasing competitive ability within riparian ecosystems.

As a nitrogen-fixing legume, *M. pigra* possesses an advantage in nitrogen-limited environments. Nevertheless, biological nitrogen fixation is energetically demanding and requires adequate supplies of complementary nutrients. The positive association observed between potassium availability and invasion density suggests that potassium may function as an important supporting resource that facilitates growth and stand development once sufficient concentrations are available.

Although plant attributes such as stem diameter often emerge as strong proximate predictors of individual plant performance, soil potassium represents a fundamental edaphic driver that can facilitate or constrain large-scale spatial expansion. While plant size reflects accrued localized growth, soil nutrient availability acts as an underlying environmental resource filter dictating habitat suitability and competitive potential across the landscape.

These findings are broadly consistent with the resource-enrichment hypothesis, which proposes that invasive alien plant species often respond more strongly to increased resource availability than native species. Under this interpretation, potassium-poor environments may partially constrain population expansion, whereas potassium-enriched microsites may provide favorable conditions for rapid establishment and increased invasion intensity.

Although exploratory texture-specific analyses suggested variation in potassium threshold estimates among soil texture classes, these patterns should be interpreted cautiously because several texture categories were represented by relatively small sample sizes. Nevertheless, the observed patterns are consistent with the ecological expectation that soil physical properties influence nutrient retention and effective nutrient availability. Differences in nutrient retention, water-holding capacity, and soil physical properties may influence the effective availability of potassium to plants. Although the underlying mechanisms were not directly measured in this study, the results suggest that soil texture may modify how nutrient availability influences invasion dynamics across heterogeneous floodplain environments.

### 4.3. Implications for Wetland Management and Restoration

The identification of a potassium-associated transition zone around 34 ppm may provide useful guidance for ecosystem monitoring and management. Rather than relying solely on vegetation surveys, management programs may benefit from incorporating soil nutrient assessments to identify locations where environmental conditions are potentially favorable for invasion.

From a restoration perspective, the results suggest that successful management of *M. pigra* may require approaches that extend beyond physical removal alone. Areas exhibiting environmental conditions associated with elevated invasion risk may remain susceptible to reinvasion following control efforts. Maintaining diverse native plant communities and restoring ecosystem function may therefore be important for increasing biotic resistance and reducing long-term invasion pressure.

More broadly, the proposed XAI–Bayesian framework provides a transparent and reproducible analytical framework for ecological assessment and environmental decision support. By combining predictive modeling with interpretable inference, it can help identify not only where invasion is likely to occur but also which environmental conditions are associated with increased invasion risk.

### 4.4. Study Limitations and Future Research

Several limitations should be acknowledged. First, the study was based on a relatively small sample size (n=50), which may have limited the detection of complex ecological interactions and reduced the precision of texture-specific transition zone estimates. Second, the identified potassium transition zones were derived from SHAP-based model responses and should therefore be interpreted as model-derived predictive indicators rather than definitive ecological boundaries. Third, the analysis was conducted within a single floodplain system, and independent external validation will be required to evaluate the generalizability of the proposed framework across broader ecological settings. Fourth, the estimated transition zones were obtained from an explainable machine-learning framework and may be influenced by modeling choices, including hyperparameter settings and smoothing specifications, although bootstrap resampling and alternative LOESS smoothing approaches produced generally consistent results. Finally, model performance was evaluated using nested internal cross-validation rather than independent external datasets. Consequently, the reported performance metrics should be interpreted as evidence of internal predictive consistency rather than definitive evidence of model transferability. Accordingly, the proposed framework should be regarded as an exploratory, hypothesis-generating approach until validated using larger, independent datasets.

Future research should incorporate larger spatial scales, repeated temporal observations, and additional environmental variables, including hydrological characteristics and nutrient cycling processes. Such studies would help refine the estimated transition zones, improve understanding of invasion dynamics, and evaluate the applicability of the proposed framework across different ecosystem types.

Overall, this study suggests that integrating explainable artificial intelligence with Bayesian inference can provide both predictive capability and ecological insight. The proposed framework offers a transparent and reproducible analytical approach for identifying nonlinear environmental responses and model-derived ecological transition zones, thereby supporting future ecological research and evidence-based invasive species management.

## 5. Conclusions

This study presents an integrated framework combining XGBoost–SHAP explainable machine learning and Bayesian Poisson regression to investigate environmental drivers associated with *Mimosa pigra* invasion in the Chi River Basin. The findings indicate that soil potassium availability, together with soil texture and plant diameter, represents an important predictor of invasion intensity within the observed environmental conditions.

The XGBoost–SHAP analysis identified a pronounced nonlinear relationship between soil potassium availability and predicted invasion density, revealing a model-derived ecological transition zone centered near 34 ppm. The LOESS-smoothed SHAP response indicated a transition point at approximately 33.98 ppm, corresponding to the change from negative to positive contributions to predicted invasion density. Bootstrap resampling further estimated a mean threshold of 32.40 ppm (95% bootstrap confidence interval: 23.88–47.64 ppm), indicating moderate uncertainty in the exact threshold location while supporting the overall stability of the nonlinear response pattern. Potassium concentrations between approximately 39 and 48 ppm exhibited the strongest positive SHAP contributions, whereas exploratory analyses suggested that soil texture may influence the effective availability of potassium and thereby modify invasion responses.

The Bayesian Poisson model independently identified a positive association between soil potassium and invasion density, providing complementary statistical evidence that was consistent with the nonlinear patterns detected by the XGBoost–SHAP analysis. Diagnostic comparisons indicated that substantial overdispersion was not evident in the observed data, supporting the suitability of the Poisson model for statistical inference.

Importantly, the identified potassium thresholds should be interpreted as model-derived indicators of invasion risk rather than fixed ecological boundaries or causal physiological thresholds. Although the proposed framework demonstrated consistent internal predictive performance under nested five-fold cross-validation, external validation using independent ecological datasets remains necessary before broader ecological application or management implementation.

Overall, the proposed XAI–Bayesian framework provides a transparent, reproducible, and interpretable analytical approach for detecting nonlinear environmental responses, quantifying associated uncertainty, and identifying ecological transition zones. Beyond the present case study, the proposed framework provides a transparent and reproducible strategy for hypothesis generation and ecological risk assessment in future studies of invasive species under diverse environmental conditions. 

## Figures and Tables

**Table 1:** Descriptive statistics of *M. pigra* density and soil physicochemical variables measured across the 50 sampling plots in the Chi River Basin, Thailand.

Variable	Mean	SD	Min	Median	Max
Response Variable					
Density (plants m^−2^)	12.49	5.81	1.50	11.50	35.50
Soil Variables					
Potassium (ppm)	46.21	14.70	21.52	50.73	70.33
Soil pH	6.61	0.81	5.00	6.52	8.48
Phosphorus (ppm)	8.06	2.84	2.18	8.43	13.10
Bulk density (g cm^−3^)	1.514	0.152	1.11	1.54	1.78

Note: Available phosphorus was determined using the Bray II extraction method. Potassium was measured using the ammonium acetate extraction method, and bulk density was determined as the oven-dry soil mass per unit volume.

**Table 2:** Comparison of predictive performance metrics and leave-one-out cross-validation between Bayesian Poisson and Negative Binomial models for estimating *M. pigra* density.

Model	Bayesian $R^{2}$	RMSE	MAE	LOOIC	elpd diff (se)
Poisson	0.522	3.77	2.78	293.6	0.0 (0.0)
Negative Binomial	0.469	3.92	2.90	299.8	−3.1 (4.2)

Note: LOOIC denotes Leave-one-out information criterion; RMSE, Root mean square error; MAE, Mean absolute error; elpd diff, Expected log pointwise predictive density difference relative to the best model; se, Standard error of the elpd difference.

**Table 3:** Posterior estimates, incidence rate ratios (IRR), and convergence diagnostics from the Bayesian Poisson model examining environmental drivers of *M. pigra* density.

Predictor	Posterior Estimate			IRR			Diagnostics	
	Mean	L-95% CrI	U-95% CrI	IRR	L-95% CrI	U-95% CrI	$\hat{R}$	ESS
Intercept	2.49	1.93	3.06	–	–	–	1.00	4147
K	0.01	0.00	0.02	1.010	1.001	1.018	1.00	4491
Clay Loam	0.57	0.26	0.87	1.786	1.302	2.391	1.00	4459
Loam	0.31	−0.57	1.13	1.491	0.567	3.107	1.00	7631
Sandy Clay	0.34	−0.04	0.69	1.422	0.962	1.985	1.00	5954
Sandy Clay Loam	0.21	−0.05	0.46	1.241	0.955	1.583	1.00	4767
Sandy Loam	0.31	−0.03	0.63	1.378	0.973	1.879	1.00	3979
Silty Clay	0.53	0.24	0.81	1.723	1.270	2.250	1.00	7601
Silty Clay Loam	−0.30	−0.96	0.27	0.775	0.382	1.316	1.00	10,083
Diameter	−0.31	−0.45	−0.18	0.737	0.640	0.834	1.00	6366

Note: CrI denotes Bayesian credible interval. IRR (Incidence Rate Ratio) values greater than 1 indicate a positive association with *M. pigra* density, whereas values below 1 indicate a negative association. ESS denotes effective sample size. All parameters exhibited $\hat{R}=1.00$, indicating satisfactory convergence.

**Table 4:** Relative importance of environmental predictors in the XGBoost model based on gain, cover, and frequency metrics.

Predictor	Gain	Cover	Frequency
Diameter	0.552	0.414	0.383
K	0.281	0.364	0.416
Texture: Clay Loam	0.046	0.017	0.014
Texture: Silty Clay	0.041	0.033	0.028
Texture: Clay	0.034	0.035	0.042
Texture: Sandy Clay Loam	0.015	0.030	0.027
Texture: Sandy Clay	0.013	0.056	0.044
Texture: Sandy Loam	0.011	0.029	0.027
Texture: Silty Clay Loam	0.005	0.020	0.014
Texture: Loam	0.002	0.002	0.004

Note: Gain represents the relative contribution of each predictor to model accuracy, Cover indicates the relative number of observations associated with the splits, and Frequency reflects how often a feature is used across decision trees.

**Table 5:** Potassium-associated transition points and ecological optimum derived from the XGBoost–SHAP analysis.

Metric	Potassium (ppm)
LOESS-derived threshold	33.98
Bootstrap mean threshold	32.40
95% bootstrap confidence interval	23.88–47.64
Ecological optimum (maximum SHAP contribution)	45.10
Range of strongest positive SHAP contribution	39.0–47.8

Note: The LOESS-derived threshold corresponds to the potassium concentration at which the smoothed SHAP response crosses zero, indicating a transition from negative to positive contribution to predicted *Mimosa pigra* density. Bootstrap estimates were obtained from 1000 resampled datasets to quantify uncertainty in threshold location. The ecological optimum represents the potassium concentration associated with the maximum positive SHAP.

**Table 6:** Soil texture-specific potassium (K) thresholds linked to positive SHAP contributions to predicted *Mimosa pigra* density.

Soil Texture	Threshold K	Median K	Sample Size	Positive SHAP
	(ppm)	(ppm)		
Clay	21.52	45.79	19	9
Sandy Clay Loam	30.51	52.81	10	4
Clay Loam	30.52	47.83	5	3
Sandy Loam	40.28	53.33	8	3
Silty Clay	55.36	58.71	3	2
Sandy Clay	61.68	61.68	3	1

Note: Threshold K represents the minimum soil potassium concentration at which SHAP values become positive, indicating a positive contribution to predicted *Mimosa pigra* density. Median K denotes the median potassium concentration among observations with positive SHAP values. Positive SHAP indicates the number of observations exhibiting positive SHAP contributions. Texture classes represented by fewer than three observations were excluded from interpretation because of insufficient sample size.

## Data Availability

The data presented in this study are available on request from the corresponding author. The datasets are not publicly available due to institutional data management and sharing policies.
